# SATB2 is a novel biomarker and therapeutic target for cancer

**DOI:** 10.1111/jcmm.15755

**Published:** 2020-09-04

**Authors:** Sanjit K. Roy, Anju Shrivastava, Sudesh Srivastav, Sharmila Shankar, Rakesh K. Srivastava

**Affiliations:** ^1^ Stanley S. Scott Cancer Center Louisiana State University Health Sciences Center New Orleans LA USA; ^2^ St. Joseph's Hospital and Medical Center Phoenix AZ USA; ^3^ Department of Biostatistics and Data Science School of Public Health and Tropical Medicine Tulane University School of Medicine New Orleans LA USA; ^4^ Department of Genetics Louisiana State University Health Sciences Center New Orleans LA USA; ^5^ John W. Deming Department of Medicine Tulane University School of Medicine New Orleans LA USA; ^6^ Southeast Louisiana Veterans Health Care System New Orleans LA USA

**Keywords:** cancer stem cells, CD133, CD24, CD44, c‐Myc, LGR5, Nanog, Oct4, pluripotency, SATB2, self‐renewal, Sox2, TCF/LEF, β‐catenin

## Abstract

Several studies have confirmed the involvement of cancer stem cells (CSC) in tumour progression, metastasis, drug resistance and cancer relapse. SATB2 (special AT‐rich binding protein‐2) acts as a transcriptional co‐factor and modulates chromatin architecture to regulate gene expression. The purpose of this review was to discuss the pathophysiological roles of SATB2 and assess whether it could be used as a therapeutic target for cancer. SATB2 modulated the expression of those genes which regulated pluripotency and self‐renewal. Overexpression of SATB2 gene in normal epithelial cells was shown to induce transformation, as a result transformed cells gained CSC’s characteristics by expressing stem cell markers and pluripotency maintaining factors, suggesting its role as an oncogene. In addition, SATB2 induced epithelial‐mesenchymal transition (EMT) and metastasis. Interestingly, the expression of SATB2 was positively correlated with the activation of β‐catenin/TCF‐LEF pathway. Furthermore, SATB2 silencing inhibited EMT and their positive regulators, and tumour growth, and suppressed the expression of stem cell markers, pluripotency maintaining factors, cell cycle and cell survival genes, and TCF/LEF targets. Based on the cancer genome atlas (TCGA) expression data and published papers, SATB2 alone or in combination with other proteins could be used a diagnostic biomarker for cancer. Although there is no pharmacological inhibitor of SATB2, studies using genetic approaches suggest that SATB2 could be a potential target for cancer treatment and prevention.

## 1 INTRODUCTION

Gene expression is tightly controlled by epigenetic regulators and transcription factors. One such factor is SATB2 (special AT‐rich binding protein‐2) which influences gene expression by regulating chromatin architecture and by acting as a transcriptional co‐factor.^1^, ^2^, ^3^, ^4^, ^5^ This gene is conserved in humans and mouse. In humans, there are three transcripts which encodes for SATB2 protein. Genetic engineering techniques have demonstrated that SATB2 knockout mice are defective in bone development and osteoblast differentiation.^2^ Furthermore, the expression of SATB2 has been associated with craniofacial patterning and osteoblast differentiation,^2^ as well as development of cortical neurons.^3^, ^4^, ^5^, ^6^

All the members of SATB family proteins have emerged as key regulators that integrate higher‐order chromatin organization resulting in tight control of gene expression. SATB2 is a newly identified gene whose function is just emerging in recent years. Its expression varies in human tissues where it is highly expressed in stem and progenitor cells. SATB2 is differentially expressed in various cancers where it plays a significant role in cancer initiation, progression and metastasis. The Cancer Genome Atlas (TCGA) data validate the higher SATB2 gene in various cancers mainly in solid tumours. Recent studies have shown that SATB2 can regulate the expression of genes necessary for cell division, cell cycle, cell proliferation, pluripotency and self‐renewal of stem cells. Like MAR‐binding proteins, it can also participate in DNA replication. Since SATB2 is highly expressed in most tumours, it can be used as a diagnostic biomarker for cancer, and its targeted inhibition can be useful for the treatment and prevention of cancer.

## 2 SATB2 REGULATES STEMNESS

Mounting evidence suggests that cancer cells are capable of possessing stem cell‐like properties which include gene expression, self‐renewal and repopulation capacities. According to cancer stem cell (CSC) hypothesis, a subpopulation of tumour cells is capable of self‐renewal and long‐term maintenance of tumours. This observation was supported by clinical trials which validated the roles of CSCs in therapeutic resistance, tumour dormancy, metastasis and cancer relapse. Self‐renewal is a process by which cells divide while maintaining the undifferentiated state. This process requires a tight control of cell cycle, and maintenance of pluripotency and self‐renewal programs. Maintaining genomic integrity is critical for generation of stem cells. When genomic integrity is compromised, mutations inappropriately activate self‐renewal programs leading to cancer development. Like stem cells, the pluripotency maintaining factors regulate self‐renewal and survival of cancer stem cells. Chromatin‐immunoprecipitation assays have confirmed the binding of SATB2 to the promoters of Nanog, Oct4, c‐Myc, Sox2 and Klf4,^7^, ^8^, ^9^ suggesting that SATB2 can act as a regulator of pluripotency and self‐renewal. Recent studies have demonstrated that SATB2 functions as a tumour promoter by enhancing the expression of c‐Myc, KLF4, Oct4, Sox2 and Nanog.^7^, ^8^, ^9^, ^10^, ^11^ Therefore, these findings enrich the mechanism of the SATB2’s oncogenic role in various cancers. However, a few questions remain to be answered. For example, other factors or regulators can act with SATB2 to regulate stemness which provides a link between tumour antigenicity, immune suppression, intratumoural heterogeneity and the resulting trajectories in human cancer. The search for other regulators will no doubt identify a novel mechanism by which dysregulation of SATB2 leads to carcinogenesis.

## 3 SATB2 REGULATES MALIGNANT TRANSFORMATION

Cellular transformation involves the transition of normal cells into the malignant state. This process is accompanied by alterations in cellular morphology, growth, and cellular function and metabolism, resulting in the acquisition of the capacity for uninhibited growth.^12^, ^13^, ^14^ SATB2 is a DNA‐binding protein that specifically binds to nuclear matrix attachment regions and plays a critical role in transcriptional regulation and chromatin remodelling.^4^, ^15^, ^16^ However, the role of SATB2 in malignant transformation of epithelial cells is not well understood. During carcinogenesis, transformed cells gain the characteristics of CSCs which play critical roles in cancer initiation, metastasis and resistance to therapy.^17^, ^18^, ^19^ We and others have demonstrated that human normal epithelial cells of several organs do not express SATB2 gene; however, it is highly expressed in cancer cell lines, CSCs and primary tumours of colon, liver, breast and pancreas.^7^, ^8^, ^9^, ^20^, ^21^, ^22^ Our recent work showed that overexpression of SATB2 in normal epithelial cells of the breast, colon and pancreas induces cellular transformation, and transformed cells gained the phenotypes of CSCs as they express stem cell markers and pluripotency maintaining factors.^7^, ^8^, ^9^ Using knockout studies, it was demonstrated that silencing of SATB2 gene in CSCs also abrogated CSC characteristics by inhibiting the markers of stem cells (CD24, LGR5, CD133 and CD44), pluripotency and self‐renewal (c‐Myc, Nanog, KLF4, c‐Myc, Oct4 and Sox2), cell cycle (cyclin D1) and cell proliferation/ apoptosis (Bcl‐2 and XIAP). These data suggest that SATB2 can regulate CSC’s population.

Using breast, pancreas, lung, colorectal and liver cancer models, we and others have demonstrated that silencing SATB2 expression by shRNA or Crisp/Cas9 significantly inhibited cell proliferation, and colony formation of various cancer cells.^7^, ^8^, ^9^, ^22^, ^23^, ^24^ Similarly, the up‐regulation of SATB2 in hepatocellular carcinoma tissues and cell lines was demonstrated by several groups, suggesting its role was oncogenic.^20^, ^22^, ^25^, ^26^ In contrary to above findings, inhibition of SATB2 expression resulted in enhanced cell growth, metastasis and poor prognosis in colorectal cancer ^27^, ^28^ and overexpression of SATB2 reduced colorectal cancer cell proliferation, invasion and migration.^29^ The reasons for this discrepancy of SATB2 function in colorectal cancer is not clear, but it could be related to the differentiation stage of the cells as gene expression profile changes in different regions of the colorectal tissues (ie proximal vs distal, and left vs right).

## 4 SATB2 REGULATES EPITHELIAL‐MESENCHYMAL TRANSITION

The epithelial‐mesenchymal transition (EMT) is a process by which epithelial cells lose their cell polarity and cell‐cell adhesion, and gain migratory and invasive behaviours to become mesenchymal stem cells through tightly regulated genetic events. EMT is essential for several developmental processes (such as mesoderm formation and neural tube formation), wound healing, fibrosis and cancer metastasis.^30^ Cells which undergo EMT up‐regulate the expression of certain proteins (N‐cadherin, vimentin) and transcription factors (Snail, Slug and Zeb1).^30^ Therefore, inhibition of EMT process could be considered as a novel therapeutic option for suppressing tumour metastasis in those cancers where death occurs mainly due to metastasis such as colorectal cancer. By comparison, the mesenchymal‐to‐epithelial transition (MET) is crucial for the formation of distant/secondary metastasis at another organ site. We and others have shown that SATB2 overexpression promotes cell motility, migration and invasion by modulating EMT‐related genes and transcription factors.^7^, ^8^, ^9^, ^11^, ^24^ In contrast, SATB2 inhibition by shRNA reverses EMT characteristics.^7^, ^8^, ^9^, ^22^ The lncRNA, SATB2‐AS1 (the antisense transcript of SATB2) inhibits cancer cell growth, EMT, and metastasis, and regulates immune response in colorectal cancer.^31^, ^32^ These studies clearly demonstrate the role of SATB2 in EMT and metastasis.

## 5 LINKING SATB2 WITH WNT/β‐CATENIN SIGNALLING PATHWAY

Although the role of SATB2 in carcinogenesis has been proposed, its mechanisms of action are not very clear. Based on limited data, questions arise whether its effects are exerted through critical cell signalling pathway. The Wnt/β‐catenin signalling pathway plays a significant role in embryonic development, cell polarity, proliferation, migration, survival, and tissue homeostasis and injury repair through generation of stem cells.^33^, ^34^ The canonical Wnt pathway is activated by the binding of a Wnt‐protein ligand to a Frizzled family receptor, leading to the formation of a larger cell surface complex with LRP5/6, which passes the biological signal to the Dishevelled protein inside the cell. E3 ubiquitin‐protein ligases ZNRF3 and RNF43 can degrade Wnt receptor complex components frizzled and LRP6 by acting as negative regulators of the Wnt pathway. R‐spondin can inhibit the activity of ZNRF3 and RNF43. Dickkopf‐1 (DKK1) can also prevent Wnt ligand from forming a complex with LRP5/6 receptors, thus acting as an antagonist. In the absence of the Wnt ligand, constitutively expressed β‐catenin is phosphorylated by CK1 and the APC/Axin/GSK‐3β‐complex, leading to ubiquitylation and proteasomal degradation of β‐catenin. Wnt ligands bind to the frizzled receptors, which cooperate with LRP5/6 co‐receptors, to initiate a phosphorylation cascade that activates dishevelled. This leads to disassociation of the β‐catenin degradation complex APC/Axin/GSK‐3β, β‐catenin stabilization and nuclear translocation, and activation of the transcription of TCF/LEF target genes.^35^, ^36^, ^37^, ^38^ In addition to Wnt binding to receptor, this pathway can also be activated by loss (eg APC, Axin2) or mutations (eg β‐catenin) of certain genes.^25^, ^39^, ^40^, ^41^ Importantly, Wnt/β‐catenin/TCF/LEF pathway also regulates those genes which are crucial for metastasis (Twist, E‐cadherin, MMP2, MMP7 and MMP9), angiogenesis (VEGF), cell cycle and cell survival (Cyclin D1 and Survivin), and pluripotency and self‐renewal of stem cells (c‐Myc, Sox2, Oct4, Nanog).

Limited studies have demonstrated the regulation of β‐catenin pathway by SATB2 in the area of carcinogenesis. In a model of colorectal cancer, the expression of SATB2 protein positively correlated with nuclear β‐catenin expression, which plays a role in poor prognosis and lower survival of cancer patients.^9^ Furthermore, inhibition of SATB2 by shRNA attenuated β‐catenin expression, TCF‐LEF transcriptional activity, and the expression of TCF‐LEF target genes such as Bcl‐2, CD44, Survivin, c‐Myc, Nanog, Cyclin D1 and XIAP in colorectal CSCs.^9^ Similarly, β‐catenin‐TCF/LEF pathway was shown to regulate pluripotency maintaining factors, stem cell markers, cell cycle and survival/ proliferation.^42^, ^43^, ^44^, ^45^ Moreover, evidences have proved that SATB2 could be a crucial factor for controlling pluripotency and self‐renewal of stem cells through β‐catenin/TCF‐LEF pathway.

## 6 REGULATION OF SATB2 BY microRNA (miRNA)

MicroRNAs (miRNAs) are small non‐coding RNA molecules 19 to 25 nucleotides that regulate post‐transcriptional silencing of target genes. A single miRNA can target hundreds of mRNAs and influence the expression of many genes often involved in a cell signalling pathways. We and others have identified certain miRNAs which regulate cancer cell proliferation, migration, invasion and metastasis by modulating the expression of SATB2. miRNA‐211, miR‐4270‐5p and miR‐449a inhibited the progression of HCC and CRC by down‐regulating SATB2 expression, and overexpression of SATB2 counteracted the inhibitory effects of these miRNAs on cell proliferation and tumour growth, suggesting the oncogenic potential of SATB2 in cancer.^20^, ^25^, ^46^ In contrast, miRNA‐182 and miRNA‐31 enhanced proliferation, EMT and metastasis of CRC by suppressing SATB2.^47^, ^48^ Low expression of the tumour suppressive miR‐34a in both HCC tissues and cell lines as compared to normal control was demonstrated.^26^ In the same study, miR‐34a inhibited cell proliferation by targeting SATB2 in HCC.^26^ In CSCs derived from human HCC, the expression of SATB2 was negatively correlated with miR34a and SATB2 rescued the miR‐34a‐mediated inhibition of CSC's viability.^22^ Other miRNAs such as miR‐31, miR‐34, miR‐182 and miR‐599 can regulate SATB2 in solid tumours mainly in breast, liver, lung and colorectal cancer. miR‐140‐5p inhibited osteogenic differentiation of human bone marrow‐derived mesenchymal stem cells by targeting SATB2, and in the same study, authors have shown that long non‐coding RNA H19 promoted osteogenic differentiation of BM‐MSCs through regulation of miR‐140‐5p/SATB2 axis.^49^ miR‐31a‐5p negatively regulated SATB2 expression and modulated the age‐related bone marrow microenvironment by regulating osteoblastic and osteoclastic differentiation,^50^ suggesting the therapeutic targeting of miR‐31a‐5p could be beneficial for osteoporosis.

## 7 EXPRESSION OF SATB2 IN CANCER

SATB2‐associated syndrome (SAS) is an autosomal‐dominant neurodevelopmental disorder caused by changes in the gene expression of SATB2.^51^ Higher expression of SATB2 gene has been reported in Merkle cell carcinoma, pancreatic, breast, colon, rectal and liver cancer patients than normal counterparts.^7^, ^8^, ^9^, ^20^, ^26^, ^52^, ^53^, ^54^, ^55^ The combination of SATB2 and CK20 was found to identify more than 95% of colorectal cancer.^52^ Another study using 50 paired colorectal and normal tissues has reported an increased expression of SATB2 in colorectal cancer tissues.^46^ Brenner tumour‐associated mucinous tumours demonstrated rare SATB2 expression and more frequently calcifications and Walthard cell nests, whereas teratoma‐associated mucinous tumours showed high SATB2 expression and RNF43 mutations and were closely resembled true gastrointestinal tumours.^56^ The appendiceal adenocarcinoma and ovarian teratomas also highly expressed CK7, CK20, CDX‐2 and SATB2.^57^ Interestingly, the combined expression of CK7 and SATB2 was able to distinguish lower gastrointestinal tumours from ovarian primary mucinous tumours.^58^ Similarly, SATB2 was highly expressed in squamous morules associated with endometrioid proliferative lesions and in the stroma of atypical polypoid adenomyoma.^59^ Higher expression of SATB2 in neuroendocrine neoplasms is restricted to well‐differentiated tumours of lower gastrointestinal tract origin and is most frequent in Merkel cell carcinoma among poorly differentiated carcinomas.^55^ SATB2 alone has shown high sensitivity and specificity in colorectal adenocarcinoma.^60^ Ma et al suggested that SATB2 and CDX2 can be used as prognostic biomarkers and for risk assessment in patients with mismatch repair protein‐deficient colorectal cancer.^61^ In breast cancer, the expression of SATB2 is significantly associated with increasing tumour grade and poorer survival.^62^ A three‐marker panel consisting of SATB2, CK20 and CDX2 was suggested for improving the detection of metastatic colorectal cancer in liver biopsy tissues.^63^ These findings suggest that SATB2 either alone or in combination with other proteins or markers can be used as diagnostic and prognostic biomarker of cancer.

## 8 CONCLUSIONS

Most of the solid tumours highly express SATB2 compared to normal tissues. Since overexpression of SATB2 induces malignant cellular transformation of epithelial cells, and these transformed cells possess properties of CSCs/progenitor cells, SATB2 could be considered as an oncogene. SATB2 alone or in combination with other proteins can be used as diagnostic biomarker marker for cancer. The expression of SATB2 was also found to activate β‐catenin pathway which is highly activated in most cancers. Inhibition of SATB2 in CSCs suppresses cell proliferation, colony and spheroid formation in suspension, and EMT characteristics. Majority of the published studies support the role of SATB2 in transformation, dedifferentiation, cancer progression and metastasis. Although there is no pharmacological inhibitor of SATB2 transcription and expression, using genetic approaches (RNAi and Crispr/Cas‐9) several studies have clearly demonstrated its oncogenic role in carcinogenesis. Interestingly, metformin reduces SATB2‐mediated osteosarcoma stem cell‐like phenotype and tumour growth through inhibition of N‐cadherin/NF‐kB signalling pathway.^64^ Future studies in transgenic mice models are needed to confirm these findings of SATB2 in cancer. Elucidation of the role of SATB2 in stemness has potential impacts in enhancing our understanding of cancer initiation, progression and metastasis. Specially in those cancers where SATB2 is highly expressed, its therapeutic targeting could be beneficial for the treatment and prevention of disease.

## CONFLICT OF INTEREST

All the authors have declared that no competing interests exist.

## AUTHOR CONTRIBUTIONS

**Sanjit K. Roy:** Conceptualization (equal); Project administration (equal); Writing‐original draft (equal); Writing‐review & editing (equal). **Anju Shrivastava:** Writing‐review & editing (equal). **Sudesh Srivastav:** Writing Review Editing (equal). **Sudesh Srivastav:** Writing Review Editing (equal). **Sharmila Shankar:** Supervision (equal); Writing‐review & editing (equal). **Rakesh K. Srivastava:** Supervision (equal); Writing‐review & editing (equal).
